# A Singapore-centric Fungal Dataset of 518 Cultivated Strains with Visual Phenotypes and Taxonomic Identity

**DOI:** 10.1038/s41597-025-06532-1

**Published:** 2026-01-22

**Authors:** Darren Wei Xian Ten, Fong Tian Wong, Yee Hwee Lim, Winston Koh

**Affiliations:** 1 Institute of Sustainability for Chemicals, Energy and Environment (ISCE2), Agency for Science, Technology and Research (A*STAR), 8 Biomedical Grove, #07-01 Neuros Building, Singapore, 138665 Republic of Singapore; 2 Singapore Integrative Biosystems and Engineering Research, Biosystems and Engineering (SIBER), Agency for Science, Technology and Research (A*STAR), 2 Fusionopolis Way, #08-01 Innovis, Singapore, 138634 Republic of Singapore; 3 Institute of Molecular and Cell Biology (IMCB), Agency for Science, Technology and Research (A*STAR), 61 Biopolis Drive, #07-06, Proteos, Singapore, 138673 Republic of Singapore; 4 Bioinformatics Institute (BII), Agency for Science, Technology and Research (A*STAR), 30 Biopolis Street, #07-01 Matrix, Singapore, 138671 Republic of Singapore

**Keywords:** Fungal genomics, Image processing

## Abstract

The fungal kingdom represents a greatly untapped resource to produce a wide range of bioactive secondary metabolites, including antibiotics, anticancer agents, industrially significant dyes and enzymes. To-date, it is estimated only less than 5% of all fungi have been characterised, a deficit that is especially pronounced in tropical regions like Singapore, where fungal diversity remains underexplored compared to northern hemisphere counterparts. This underlines the urgency and importance of our research which motivated the creation of our curated dataset, aiming to address this gap and contribute to understanding the broader ecosystem. We developed a generalisable cultivation workflow that enables systematic strain preparation, supports high-resolution imaging, and yields sufficient fungal biomass amenable for genomic analyses. This resulted in a diverse collection of 518 phylogenetically and ecologically varied fungal strains from both terrestrial and marine environments in biodiverse Singapore. The curated dataset from this project captures both taxonomic identity and colony-level morphological traits serving as a foundation for visual phenotype to taxonomy mapping through the integration of computer vision.

## Background & Summary

Fungi comprise one of the most diverse and ecological indispensable kingdoms on Earth. They are deeply integrated into the evolutionary history of life and continually serve as primary decomposers, facilitating nutrient cycling that sustains ecosystems^1–3^. As one of nature’s most prolific chemists, fungi synthesise an extraordinary repertoire of molecules, with transformative applications across medicine, biotechnology and industry^4–7^. The vast potential and opportunities offered by fungal chemistry has pushed efforts to broaden the exploration of fungal diversity, as exemplified by several large-scale initiatives. Notably, the Joint Genome Institute (JGI)’s MycoCosm’s platform currently hosts over 2,500 publicly available fungal genomes, compiled through a combination of community-contributed datasets and in-house sequencing efforts. This is primarily from the 1000 Fungal Genomes Project, which prioritises sequencing of reference genomes across more than 500 fungal families^8,9^. Another major initiative is MycoBank, a global database and inter-repository hub for the registration and standardisation of novel fungal species nomenclature, also providing reference access to associated fungal taxonomic and genomic resources^10,11^.

Despite this global momentum to elucidate the projected existence of 5 to 12 million fungal species, only ~150,000 species have been formally described, with an even smaller proportion explored for their biotechnological potential^12–15^. This disparity is particularly pronounced in tropical regions, where characterized fungi species are predominantly from the northern hemisphere^16,17^. Situated in the biodiverse equatorial region of Southeast Asia, Singapore harbours a remarkable diversity of flora and fauna, with an estimated 40,000 species, with a recent biodiversity survey formally documented over 3,000 species^18,19^, positioning it as a haven for concentrated biodiversity research. Comparatively, more studies can be emphasised on Singapore’s fungal biodiversity, with much of its ecology and functional properties remained unexplored, hampering efforts on further biotechnological utilisation, conservation, and understanding of the broader ecosystem^20–22^.

To address this imbalance, we implemented a rapid, streamlined and scalable solid-state fermentation workflow that bins collected isolates by terrestrial and marine origins and employed two general purpose media, identified for its ability to support robust biomass production across phylogenetically diverse lineages. Applied to a curated collection of 1,136 cryopreserved fungal isolates, this workflow achieved an 80.2% revival rate (n = 911; 99% CI: 77.1%-83.2%). High-resolution images were also collected across 3 key culture milestones [Day 7, Day 14, and pre-harvest (Day 11 to Day 71)] for some of the strains. Of the successful revived isolates, 94.1% (n = 857; 99% CI: 92.3%-95.9%) yielded sufficient biomass for DNA extraction, facilitating taxonomy curation with 18S. Quality assurances involved decontamination screening to omit non-fungal sequences and exclusion of fungal genomes lacking pre-harvest colony images.

**Fig. 1:**
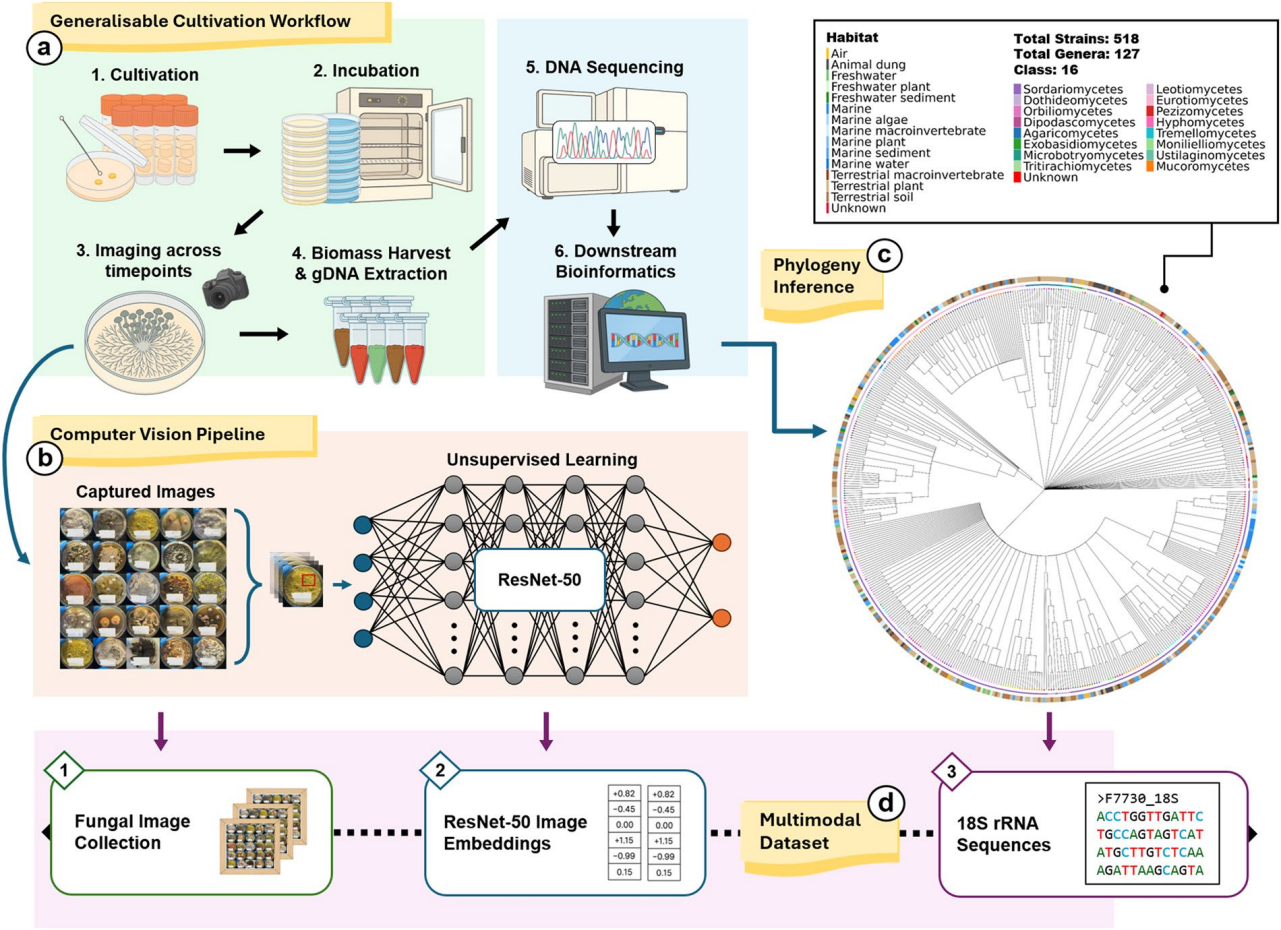
Overview of the multi-modal fungal dataset, providing an integrated perspective into the phenotypic and genomic diversity of Singapore’s fungi. (**a**) The cultivation workflow involves the following steps: First, (1) cryopreserved isolates are revived through solid-state cultivation and (2) incubated under standardised conditions, with (3) high-resolution colony images captured at key developmental milestones. Once colonies reach target biomass, samples underwent (4) DNA extraction, library preparation, and sequencing. (**b**) Computer vision pipeline for the processing of fungal colony images to extract high-dimensional feature embeddings. (**c**) Phylogenetic tree of 518 fungal strains annotated with class‐level taxonomy and habitat origin. (**d**) Generated multimodal dataset consisting of (1) fungal image collection across key development milestones, (2) ResNet-50 embeddings of all images, and (3) 18S rRNA sequences for taxonomic inference.

The eventual curated collection comprises 518 diverse fungal strains, each paired with colony morphology images across various timepoints amounting to a total of 1,528 images. We processed the 518 fungal 18S rRNA sequences with multiple alignment using fast Fourier transform (MAFFT) to produce uniform base pair alignments^23^. With this alignment, we used FastTree to infer a maximum-likelihood phylogeny^24^, applied 100 bootstraps to obtain a majority-rule consensus tree, followed by visualisation with GGTREE^25^. Annotated with taxonomic assignments and ecological metadata, the phylogenetic tree offers a comprehensive overview of Singapore’s fungal diversity. Spanning 127 genera and 3 unassigned strains across 16 classes and 4 phyla, the dataset represents fungal strains from a broad spectrum of habitats across the island, from soils and freshwater systems to marine environments (Fig. 1; Table 1).

**Table 1:** Taxonomic and Ecological Distribution of 518 Singapore Fungal Strains.

Taxonomic Distribution		Ecological Distribution	
Description	Count	Description	Count
Total Genera	**127**	Terrestrial Habitat	316 (61.0%)
Highest Represented Genus (*Fusarium*)	56 (10.8%)	Marine Habitat	134 (25.9%)
Species with Unknown Lineages	3 (0.6%)	Animal Dung Habitat	35 (6.8%)
2–10% Genera (*Hypocrea, Penicillium, Purpureocillium, Aspergillus, Verticillium, Phoma, Leptosphaeria, Hypoxylon, Proxiovicillium, Myrothecium, Cochliobolus*)	216 (41.7%)	Freshwater Habitat	27 (5.2%)
Others	243 (46.9%)	Others	6 (1.2%)

**Fig. 2:**
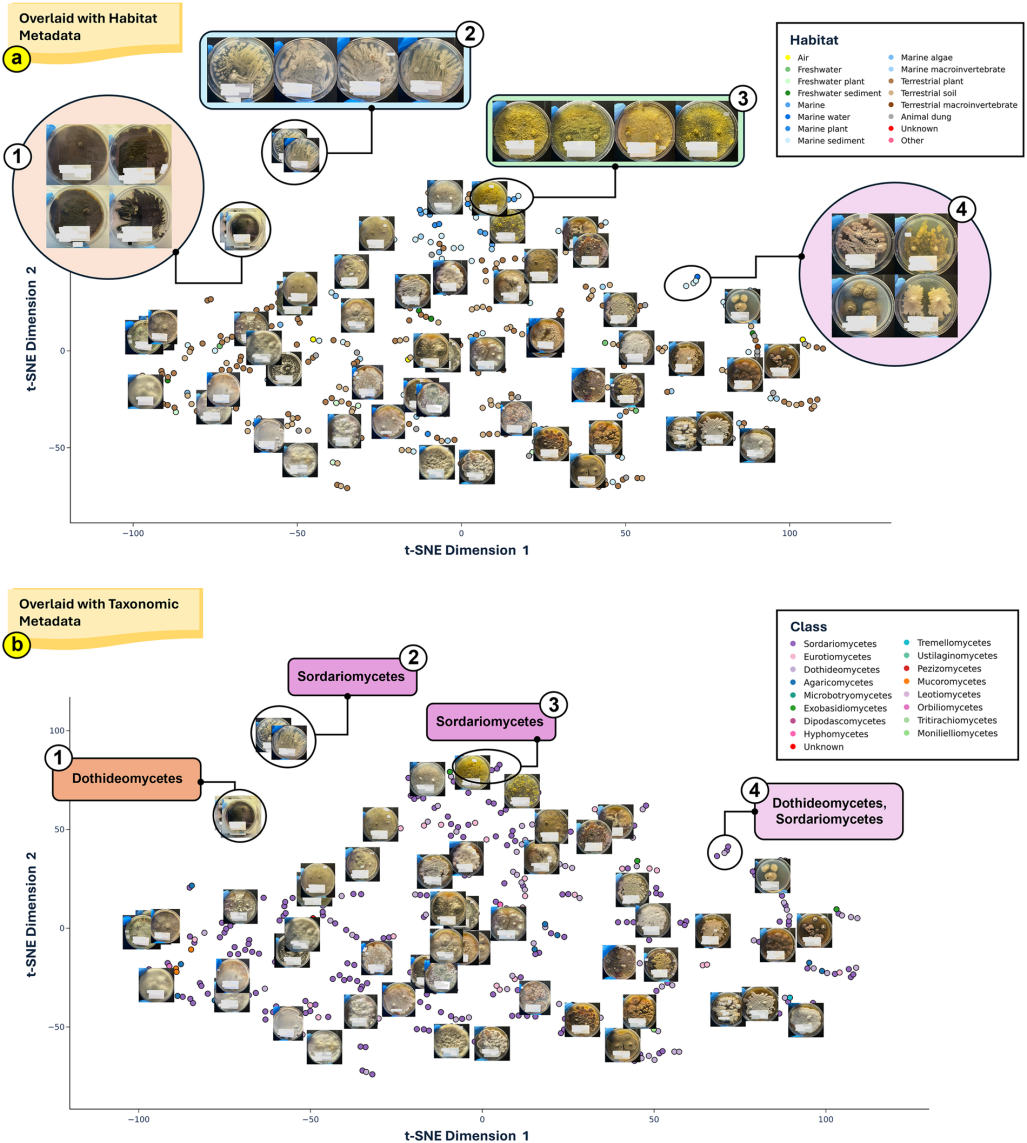
t-SNE projections of 518 ResNet-50 derived feature embeddings from pre-harvest fungal colony images, facilitating phenotypic-genomic analysis. Embeddings (points) are coloured with different metadata, facilitating phenotypic-genomic analysis. (**a**) Embeddings coloured according to the isolate’s habitat of origin, with emphasis on four key clusters with distinct morphotypes: (1) deep-black strains, (2) clear-white strains, (3) yellow strains, and (4) circular and lobate colony morphology. (**b**) The same embedding space as (**a**), coloured with class-level taxonomy, highlighting the same four clusters.

To demonstrate the utility of the multi-modal dataset, we focused on 518 pre-harvest colony images drawn from the complete image collection of 1,528. High-dimensional representations of image features of the strain collection were extracted using a Residual Network with 50 layers (ResNet-50) convolutional neural network (CNN)^26^. Prior to feature extraction, input images were pre-processed with the Efficient and Accurate Scene Text (EAST) detection model to identify and occlude text regions^27^, ensuring downstream analyses were driven solely by morphological traits. The resulting image embeddings effectively captured ecological niches, providing a direct linkage between phenotype and taxonomy. To evaluate the efficacies of the captured images and their corresponding embeddings in preserving taxonomic resolution and ecological niches, we projected the 2048-dimensional embeddings into two-dimensions using t-distributed stochastic neighbour embedding (t-SNE) (Fig. 2). Overlaid ecological metadata and captured pre-harvest images, this projection (Fig. 2a) revealed interesting phenotypic clusters, most notably by four coherent clusters defined by pigmentation: (2a-1) strains from diverse habitats exhibiting a deep black phenotype; (2a-2) clear white phenotypes from freshwater and marine sources; (2a-3) yellow strains predominantly from marine sources. Morphology-based clustering was also evident, highlighted by a cluster of strains displaying circular and lobate colony morphotype (Fig. 2a-4). Alternative overlaying with taxonomical metadata enabled seamless toggling between phenotype and taxonomy context (Fig. 2b). For example, the black phenotype cluster (Fig. 2a-1) corresponds to Dothideomycetes genera such as *Hortaea* and *Cochliobolus* (Fig. 2b-1), both documented for their dark colony morphology^28,29^. This first-pass analysis validates our computer vision pipeline and further demonstrates the utility of the multimodal dataset to capture meaningful biological nuances.

Beyond these exploratory findings, this dataset provides not only a streamlined cultivation workflow adaptable to a wide spectrum of fungal taxa but also presents a unique opportunity to interrogate the distinct colony morphology and underlying genomic signatures of 518 fungal strains from Singapore. It includes time-stamped fungal images across key developmental milestones, high-dimensional ResNet-50 embeddings, predicted 18S rRNA sequences, and corresponding taxonomy and ecological metadata. Together, these components form a unified platform for bridging visual phenotype to taxonomy, serving both as a reference for fungal biodiversity in tropical Singapore and as a hypothesis-generation platform, spurring targeted future investigations and supporting downstream genomic exploration for deep phenotypic and taxonomic insights.

The current dataset provides valuable insights into fungal diversity through 18S sequencing and representative time-point imaging, enabling analysis of species-level patterns. While we recognise that distinguishing closely related taxa may benefit from additional data dimensions, this foundational approach significantly advances our understanding of fungal ecological dynamics. Incorporating high-confidence whole-genome assemblies and expanded temporal imaging in future studies will further improve taxonomic accuracy, uncover new morphotype patterns, and elucidate the molecular mechanisms driving phenotypic variation.

## Methods

### Cultivation, imaging and harvest

A total of 1,136 uncharacterized fungal isolates from Singapore was cultivated. These isolates are part of the Natural Product Library (NPL) collection in Agency for Science, Technology and Research (A*STAR)^30^. Cryovials containing preservation fluid (reverse osmosis water with 25% glycerol) and fungal agar plugs (agar with fungal mycelium) were retrieved from a −80 °C freezer and allowed to thaw completely at room temperature. Cryopreserved fungal plugs were cultured on either ME agar [malt extract (30 g/L), agar (15 g/L), mycological peptone (5 g/L)] or GYS agar [sea salt (40 g/L), agar (15 g/L), glucose (10 g/L), yeast extract (1 g/L)], selected based on terrestrial or marine origin. Cultures were incubated at 25 °C (42 °C for thermophilic isolates), and colony morphology was photographed in a biosafety cabinet across key developmental stages (varied based on growth rate) using an iPhone 15 Pro (48-megapixel, macro mode).

### DNA sequencing, taxonomic inference and phylogenetic analysis

Fungal DNA was extracted from biomass and sequenced on the Illumina NovaSeq 6000 platform. 18S regions were predicted with (Barrnap, v0.9), applying eukaryote-specific hidden Markov model (HMM)^31^. To facilitate taxonomic assignments at a genus level, the predicted 18S regions of 518 strains were queried against NCBI core nucleotide database (accessed on 24 April 2025) with BLASTn (v2.14.1). Upon filtering by bit-score, each strain was provisionally assigned to the genus corresponding to its top BLAST hit, further supplemented with phylum, sub-phylum, and class assignments with records from JGI’s MycoCosm (accessed on 4 September 2024) and MycoBank database (accessed on 1 June 2025).

Phylogenetic tree was constructed by performing multiple sequence alignment (MSA) on the 518 fungal 18S rRNA sequences using MAFFT (v7.525) using the–auto mode (FFT-NS-2 algorithm)^23^. The resulting alignment consisted of 518 sequences with equal length (2,287 bp). Phylogenetic robustness was assessed with 100 bootstrap replicates (resampled alignment columns with replacement) and a maximum-likelihood trees was reconstructed with FastTree 2 (v2.1.11)^24^, employing the Generalized Time Reversible (GTR) model with gamma-distributed site-rate variation. Clade support values were derived by generating a majority-rule consensus tree using the R-package Analyses of Phylogenetics and Evolution package (ape, v5.8.1)^32^, with clades retained if supported in ≥50% of replicates (518 tips, 262 internal nodes, mean bootstrap support of 84%). The tree was visualised with GGTREE (Bioconductor v3.2)^25^, annotated with genus, class, and habitat metadata.

### Image Pre-processing, ResNet-50, and t-SNE Visualisation

The 518 pre-harvest fungal colony images were first converted to.jpg format, resized and padded to 1024 pixel while preserving their aspect ratio, ensuring dimensional compatibility with the frozen EAST text detection model^27^. The pretrained frozen EAST text detection model (inference-optimised) generated geometry maps and confidence scores for detected text presence, subsequently decoded into bounding boxes by translating model-predicted dimensions into input-image coordinates. Bounding boxes with confidence scores below 0.2 were discarded, retaining text regions within a generous confidence range. To mask these detected text regions and minimise bias in downstream phenotypic analysis, a Gaussian blur and semi-transparent overlay were applied to each bounding box.

The masked images were subsequently resized to a resolution of 224 × 224 pixels to enable feature extraction by the ResNet-50 model. The images were then transformed into PyTorch tensors, converting pixel values to normalised floats in [Channel, Height, Width] format. These tensors were standardised using ImageNet dataset parameters (channel-wise mean [0.485, 0.456, 0.406], standard deviation [0.229, 0.224, 0.225]), aligning with ResNet-50 originally training conditions^26^. These vectors were iteratively fed into the ResNet-50 model for unsupervised feature extraction, extracting 2048-dimensional embeddings for each input image from the final average pooling layer of the model. Principal component analysis (PCA) and t-SNE, implemented via scikit-learn (v1.6.1), were applied to the 518 image embeddings to denoised the original high-dimensional feature space to 10 principal components, followed by further dimensionality reduction to two-dimensions (n_components = 2, perplexity = 5, random state = 42). Relevant metadata (e.g., taxonomy, ecological origin) were overlaid onto the t-SNE plot to guide interpretation of the ResNet-50-derived image clusters.

## Data Records

The 518 fungal dataset comprises of (1) fungal colony morphology images across key developmental stages (.jpg format), (2) ResNet-50 pre-harvest image embeddings (PyTorch format), (3) fungal strains taxonomy and ecological metadata, and (4) Barrnap-extracted 18S region (.fasta format). All components of the dataset are available at Figshare (10.6084/m9.figshare.29434469.v2)^33^. The 18S sequences have also been deposited and is publicly accessible in the NCBI Sequence Read Archive (SRA) under the accession number SRP647581 (https://identifiers.org/ncbi/insdc.sra:SRP647581)^34^.

## Technical Validation

Captured fungal colony images were first subjected to automated text occlusion by the frozen EAST text detection model, followed by processing with the ResNet-50 CNN model. The extracted 2048-dimensional embeddings capture ecological and taxonomical features, highlighting the robustness of the dataset.

## Usage Notes

This dataset enables users to investigate underexplored fungal lineages and help address gaps in the fungal phylogeny, with emphasis on Singapore’s rich mycobiota. Additionally, this dataset provides the unique opportunity to examine the dynamic colony morphology of 518 strains and explore its relationship to corresponding genomic signatures, presenting a valuable atlas for visual phenotype to taxonomic linkage. Accompanied by detailed strain metadata, the dataset, alongside Resnet-50 derived image embeddings and clustering, functions as a rapid hypothesis-generation platform. Users can identify fungal clusters of interest based on its phenotypic characteristics and rapidly relate its underlying genomics, creating a systematic and targeted exploration strategy for downstream functional or genomic studies. This phenotypic-taxonomic framework is readily amenable to additional visual and language model applications, to extract deeper insights from the rich mine of visual and genomic data.

## Data Availability

All dataset components have been made publicly available on Figshare^33^. The 18S sequences have also been deposited to the NCBI SRA under the accession number SRP647581 (https://identifiers.org/ncbi/insdc.sra:SRP647581)^34^.

## Code availability

Scripts for (1) 18S rRNA prediction with Barrnap, (2) MAFFT alignment, (3) FastTree maximum-likelihood tree inference, (4) consensus tree construction and (5) ResNet-50 embedding generation are available on the GitHub repository (https://github.com/twxdarren/fungal-trove.git) and Figshare^33^.
